# From mold to mycotoxins: an LC–MS/MS method for quantifying airborne mycotoxins in indoor environments

**DOI:** 10.1007/s00216-025-05980-3

**Published:** 2025-07-23

**Authors:** Wiebke Derz, Paul W. Elsinghorst

**Affiliations:** 1 Central Institute of the Bundeswehr Medical Service Munich, Ingolstädter Landstraße 102, 85748 Garching, Germany; 2 Institute of Nutrition and Food Sciences, University of Bonn, Friedrich-Hirzebruch-Allee 7, 53115 Bonn, Germany; 3 Present Address: German Federal Ministry of Defence, Stauffenbergstraße 18, 10785 Berlin, Germany

**Keywords:** Indoor mold, Total suspended particulate matter (TSP), Dust sampling, Mycotoxins, Mycotoxicosis

## Abstract

Airborne exposure to mycotoxins and fungal spores poses a significant yet often overlooked risk to human health. Inhalation and ingestion of mycotoxins being adsorbed onto dust particles or as part of mycelium debris are the primary pathways of incorporation. Exposure assessment, however, is hampered as the detection of airborne mycotoxins remains challenging due to very low sampling masses, ultra-trace level toxin concentrations, and the complexity of dust matrices. Here, we present a reliable sampling and LC–MS/MS-based quantification method for 29 mycotoxins highly relevant to airborne mycotoxin exposure. Total suspended particulate matter (TSP) was collected using a stationary sampler equipped with glass fiber filters, which were subsequently classified according to composition and extent of their loading. Extraction conditions were optimized and standard addition applied to match the observed matrix effects. The method was fully validated by applying a paired observations approach covering working ranges and quantification limits applicable to ultra-trace analysis. Applicability of the method has been demonstrated in three authentic cases of mold-infested working environments, where seven different mycotoxins including alternariol monomethyl ether, sterigmatocystin, and stachybotrylactam were successfully detected and quantified.

**Graphical abstract:**

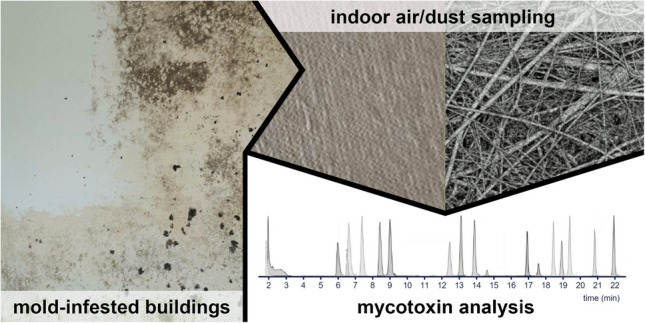

**Supplementary Information:**

The online version contains supplementary material available at 10.1007/s00216-025-05980-3.

## Introduction

The World Health Organization (WHO) guidelines on indoor air quality emphasize the health risks associated with mold exposure—an issue well recognized by the public [1]. With the increasing frequency of heavy rainfall and flooding events due to climate change, the likelihood of encountering indoor mold is growing. In these cases, the inevitable question arises if the situation poses a serious risk to human health. Answering this question is, however, not a simple task. Inhalation of pathogenic fungi, their spores, mycotoxins, and other secondary metabolites is associated with the development of various illnesses, with asthma and pulmonary or even systemic mycosis being prominent examples [1–5]. In particular, the mycotoxins produced by many mold species can pose a significant threat to human health and require appropriate attention [6, 7]. It is therefore necessary to assess both, exposure to vital fungi or spores and to mycotoxins. This study focuses on the latter.

Mycotoxins can become airborne either actively through the fungi releasing mycelium debris and spores or passively via secreted exudates, which can adhere to total suspended particulate matter (TSP). The variety of mycotoxins, being fungal secondary metabolites, is enormous, be it in number, chemical structure, or toxic effects, and as such already hard to grasp. In addition, their formation through fungal metabolism is complex and depends on many different factors such as ambient temperature, available substrate, or sequences of climatic events [8, 9]. Extremely wet periods providing beneficial growth conditions followed by dry periods may cause stress to the fungi triggering spore and mycotoxin production. However, the general ability of a mold to produce a mycotoxin does not automatically mean it will actually be formed in the event of an infestation [10]. A possible exposure can therefore only be evaluated by sampling indoor air, settled dust, or the infested wall material. Sampling of wall material usually requires a defined thickness, which, depending on the nature of the wall, can be a complicated task. By contrast, settled dust can be more easily collected from furniture or carpets as well as vacuum cleaner bags, while airborne dust from indoor air is typically captured by either equipping a person with a portable collector or by monitoring an entire room using a stationary sampling device. The major advantage of sampling indoor air compared to the other approaches is the additional information obtained on respiratory exposure. In this work, we therefore used a stationary sampling device to collect all kinds of airborne particles like spores, mycelium debris, and exudate-covered dust particles from isolated mold-infested rooms.

Typical molds infesting buildings and producing a broad spectrum of mycotoxins are various *Aspergillus* and *Penicillium* species as well as *Stachybotrys* spp., *Chaetomium* spp., and *Alternaria alternata* [11]. Some research has been reported in recent years on the detection of mycotoxins in indoor air, particularly with regard to *Stachybotrys chartarum* trichothecenes that have been related to the phenomenon of sick building syndrome [12]. Especially in water-damaged buildings across Europe and North America, macrocyclic trichothecenes could be detected in indoor air (Table 1) [13–16]. However, exposure data is still sparse and some toxins like *Alternaria* toxins have not been studied in indoor air at all. Additionally, considering warming climates, the spectrum of fungi and resulting mycotoxins is very likely to shift; e.g., an increased occurrence of aflatoxins is expected particularly in Europe as *Aspergillus flavus* spreads [17]. Currently only common to food, it has already been isolated from indoor air and may in turn lead to a future increase in airborne aflatoxins [18].

**Table 1:** Mycotoxins detected in indoor air/airborne dust of water-damaged buildings across Europe and North America

Country	Sample origin	Sampling technique	Analytical method	Detected toxins (ng/m^3^)	References
Germany	1 water-damaged building	Stationary sampler Sampling time: 15 h Flow rate: 5 m^**3**^/h	LC–MS/MS	Satratoxin G: 0.25 Satratoxin H: 0.43	[13]
France	15 *Stachybotrys*-infested dwellings	Stationary sampler Sampling time: 4 h Flow rate: 23 L/min	ELISA	Macrocyclic trichothecenes: 0.29–0.62	[14]
Belgium	7 water-damaged buildings	Stationary sampler Sampling time: 3.5 h Flow rate: 200 L/min	LC–MS/MS	Roquefortin C: 1.0–4.0 Chaetoglobosin A: 0.0067–3.4 Sterigmatocystin: 0.0034–1.77 Aflatoxin B_1_: 0.0024–0.15 Aflatoxin B_2_: 0.0003–0.02 Roridin E: 0.0031–0.08 Ochratoxin A: 0.01–0.23	[15]
USA	7 *Stachybotrys*-infested buildings	Stationary sampler Sampling time: ≤ 120 min Flow rate: 450 L/min	ELISA	Macrocyclic trichothecenes: < 0.01 to > 1.3	[16]

A set of 29 toxicologically relevant and commercially available mycotoxins associated with mold-infested buildings was selected for the development of an indoor air/TSP sampling and LC–MS/MS-based quantification method to assess indoor air exposure (Table 2). Three representative use cases are included to serve as a proof-of-concept.

**Table 2:** Mycotoxins included based on their relevance to indoor environments [6, 11, 19], toxicological effects, and commercial availability

	Analyte	Fungal origin	Toxicological effects	References
AFB1	Aflatoxin B_1_	*Aspergillus flavus*, *Aspergillus parasiticus*, *Arthrinium* spp.	Genotoxic, mutagenic, teratogenic, carcinogenic (IARC Group 1), immunosuppressive, nephrotoxic, and cytotoxic effects	[20]
AFB2	Aflatoxin B_2_			
AFG1	Aflatoxin G_1_			
AFG2	Aflatoxin G_2_			
AME	Alternariol mono-methyl ether	*Alternaria alternata*	Putative etiological factor for esophageal cancer (AME, AOH); endocrine-disruptive and immunotoxic effects in vitro (AOH, AME); genotoxic effects in vivo (AME); cytotoxic effects in vitro (AOH, TA); mutagenic effects in vitro (AOH, AME); hemorrhagic in dogs and toxic to animals (TA)	[21]
AOH	Alternariol			
TA	Tenuazonic acid			
FUM	Fumagillin	*Aspergillus fumigatus*	Clastogenic activity in human lymphocytes, antiproliferative, and genotoxic potential in mice (FUM); tremorgenic, genotoxic, neurotoxic, and mutagenic effects in vitro (FTB, FTC, VER); immunosuppressive and cytotoxic effects (GTX)	[22–25]
FTB	Fumitremorgin B			
FTC	Fumitremorgin C			
GTX	Gliotoxin			
VER	Verruculogen			
RE	Roridin E	*Stachybotrys* ssp.	Highly cytotoxic macrocyclic trichothecenes (RE, RL2, STG, STH, VRA); immunotoxic and neurotoxic phenylspirodrimane, and endothelin antagonist (SBL); cytotoxic effects in vitro and hemorrhagic in lambs (VRA)	[9, 26–28]
RL2	Roridin L2			
SBL	Stachybotrylactam			
STG	Satratoxin G			
STH	Satratoxin H			
VRA	Verrucarin A			
MPA	Mycophenolic acid	*Penicillium* ssp.	Immunosuppressive and toxic to the gastrointestinal tract (MPA); neurotoxic to cockerels (RQC); often co-occurring showing synergistic effects (MPA, RQC)	[29–31]
RQC	Roquefortin C			
CGA	Chaetoglobosin A	*Chaetomium globosum*	Highly cytotoxic effects in vitro	[32, 33]
CTV	Citreoviridin	*Penicillium citreonigrum*, *Aspergillus terreus*	Respiratory and cardiovascular failure, systemic hypoxia, and central nervous system depression at high doses	[34]
CIT	Citrinin	*Monascus ruber*, *Penicillium* ssp.	Potential carcinogenicity (IARC group 3), contradictory data to carcinogenic effects in mice, cytotoxicity and nephrotoxicity in animals, and reproductive toxicity in rats	[35, 36]
CPA	Cyclopiazonic acid	*Aspergillus* ssp*.,* *Penicillium* ssp.	Degenerative effects and multiple organ necrosis in rats, cytotoxic in vitro, and additive effects with aflatoxins	[37]
OTA	Ochratoxin A	*Aspergillus* ssp., *Penicillium* ssp.	Possibly carcinogenic (IARC group 2B), nephrotoxic, hepatotoxic, teratogenic, and immunotoxic effects	[38]
PA	Penitrem A	*Penicillium* ssp.	Tremorgenic in cockerels	[30]
RUG	Rugulosin	*Talaromyces* spp.	Potential carcinogenicity (IARC group 3); (+)-RUG exhibits DNA interaction in vitro; hepatotoxic and hepatocarcinogenic effects in mice	[39, 40]
SAF	Secalonic acid F	*Penicillium chrysogenum*	Cytotoxic effects in vitro	[41]
STC	Sterigmatocystin	*Aspergillus versicolor*	Possibly carcinogenic (IARC group 2B); putative etiological factor for gastric and hepatocellular carcinoma, hepatotoxic and nephrotoxic effects in animals, and carcinogenic in rodents and monkeys	[42]

## Materials and methods

### Chemicals

All solvents and reagents used for chromatography were of LC–MS grade quality. Methanol and 2-propanol were purchased from Riedel-de-Haën (Seelze, Germany). Acetonitrile was derived from VWR (Darmstadt, Germany). Formic acid, ammonium formate, and ammonia (25% aq.) were obtained from Fluka (Buchs, Switzerland). Water was prepared in-house using a Milli-Q Gradient water purification system from Merck (Darmstadt, Germany).

AFB1, AFB2, AFG1, and AFG2 were obtained from LGC Standards (Augsburg, Germany). AME, AOH, and OTA were purchased from HPC Standards (Borsdorf, Germany). CGA, FUM, RQC, RE, and VRA were derived from Cfm Oskar Tropitzsch (Marktredwitz, Germany). CIT was purchased from Sigma-Aldrich (Taufkirchen, Germany). CTV, CPA, STC, and TA were obtained from Santa Cruz (Dallas, TX). FTB, FTC, GTX, MPA, PA, RL2, RUF, STG, STH, SAF, SBL, and VER were purchased from Biomol (Hamburg, Germany).

Stock solutions were prepared using methanol or according to the manufacturer’s instructions. When no stability information in solution was available, aliquots were transferred to silanized amber vials, evaporated to dryness, and flushed with nitrogen for later reconstitution. Vials were stored at –80 °C.

### Total suspended particulate matter (TSP) sampling

While matrix blank samples for method development were collected from a dry, apparently mold/odor-free storage room with no detectable mycotoxin levels, mold-infested working environments usually experiencing intensive physical usage were sampled to evaluate the method’s applicability (see Electronic Supplementary Material, Section S-1 for photos of the sampling sites). To simulate worst-case inhalation exposure, doors and windows were tightly closed and settled dust inside the sampled rooms was put in motion prior to sampling (see Electronic Supplementary Material S-2 for further details). TSP was collected after 30 min using an MVS 6.1 air sampler (Comde-Derenda, Stahnsdorf, Germany) using glass fiber filters (Ø 47 mm, Whatman GF/A, VWR). Sampling conditions (pressure, temperature, air flow) were continuously monitored providing reference to Nm^3^/h as given by ISO 2533 (1 Nm^3^ equals to the volume of 1 m^3^ at 15 °C and 1013.25 hPa) [43]. Based on site-specific conditions (e.g., available total room volume and sampling time), samples were collected for 3 to 10 h at flow rates of 2.3 to 4.6 Nm^3^/h. Loaded filters were carefully removed from the sampling head, placed in small petri dishes, sealed with adhesive tape, and stored at –80 °C until extraction.

### Filter characterization

Qualitative and semi-quantitative analyses of blank matrix filter loadings were based on representative punched out circles (0.07 cm^2^) subjected to scanning electron microscopy (SEM) using electron backscatter diffraction (EBSD) to distinguish between organic and inorganic particles (Zeiss Ultra Plus, Oxford Ultim Extreme; Zeiss, Oberkochen, Germany). Energy-dispersive X-ray (EDX) analysis was applied to map element distribution at 50 × and 500 × magnification. Quantitative analysis of filter loadings was subsequently carried out using a reflected-light microscope equipped with a polarization filter (BX53M, Olympus, Hamburg, Germany). Mapping of representative strips (1.2 cm^2^) of the filter surface at 50 × magnification provided mappings that were converted to 256-level grayscale histograms using ImageJ (version 1.54f) for classification [44]. Corresponding pixel counts were summed up, normalized with respect to the total pixel count, and compared to the most densely loaded filter assigned a reference loading of 100%.

### Extraction experiments

Extraction conditions were optimized based on recovery experiments spiking unexposed as well as two matrix blank filters (moderately and heavily loaded). Methanol/2-propanol (9/1, v/v) as the organic phase applied for chromatography was used to evaluate extraction at five different time points (30, 60, 90, 120, and 240 min) in six replicates. To minimize variability resulting from differences in filter appearance and matrix load, a single filter was used per experiment and evenly split into six parts. Filter parts were then placed in safe-lock reaction tubes (5 mL), followed by addition of standard solution (100 µL, 0.2–275 pg/µL, i.e., up to 10 × LOQ providing suitable LC–MS/MS signals) and extraction solution (400 µL). Sonication at 20 °C was applied during the following extraction and at each time point, a brief centrifugation step (10 s, 1000 rpm, 96 × *g*) was applied to collect any droplets before an aliquot (10 µL) was taken. After dilution with additional extraction solution (20 µL), the mixture was applied to 0.45 µm PTFE micro spin filters, centrifuged (3 s, 13,000 rpm, 16,278 × *g*) and transferred to silanized inserts. Final mixing in separate vials of the extract (6 µL) with the respective aqueous eluent (14 µL) for chromatography was accomplished using the autosampler before injection of 10 µL. Recoveries normalized to standard concentrations in methanol/2-propanol (9/1, v/v) were visualized using gnuplot (version 5.4) [45].

### Sample preparation

For routine analysis, sample collection filters were extracted using methanol/2-propanol (9/1, v/v; 3 mL) in tightly sealed PP urine beakers of suitable diameter allowing for complete immersion. Sonication (20 °C, 30 min) was applied, followed by a brief centrifugation step (10 s, 1000 rpm, 209 × *g*) to collect any droplets from the beaker wall and lid. Extracts were subsequently applied to 0.45 µm PTFE micro spin filters, centrifuged (10 s, 13,000 rpm, 16,278 × *g*), and transferred to silanized amber vials. Extracts were finally mixed in-needle with the respective eluent and six-point equidistant calibration curves were automatically prepared for quantification by standard addition using the autosampler prior to injection. Spiking levels were individually adjusted to match the 1- to fivefold of the analyte concentration estimated in a preliminary screening (see Electronic Supplementary Material, Section S-3 for further details on the applied autosampler program).

### LC–MS/MS analysis

Chromatographic analysis was carried out using an Infinity 1260 series chromatography system (Agilent Technologies, Waldbronn, Germany) consisting of an autosampler, a binary pump, and a column thermostat, which was coupled to a 6470 triple quadrupole mass spectrometer equipped with an Agilent Jet Stream ion source (see Electronic Supplementary Material, Section S-4 for further details). Data acquisition, processing, and analysis for LC–MS/MS were performed using MassHunter Optimizer and Workstation (version 10.0, Agilent Technologies). Chromatographic separation was achieved using two different settings.

Conditions A (AFB1, AFB2, AFG1, AFG2, AOH, AME, CPA, FUM, FTB, FTC, GTX, PA, RQC, RE, STG, STH, SBL, STC, TA, VER): column Phenomenex (Aschaffenburg, Germany) Gemini NX-C18 (150 × 3 mm, 3 µm), gradient elution (A: 5 mM ammonium formate, 0.3% ammonia, pH 10, B: methanol/2-propanol, 9/1, v/v; min-%B-flow) 0–30-0.4, 0.2–40-0.4, 9–50-0.4, 10–60-0.4, 19–80-0.4, 20–80-0.4, 21–100-0.56, 22.7–100-0.56, 23–30-0.56, 38.5–30-0.56, 38.6–30-0.4, 40–30-0.4, 40 °C, injection volume 30 µL.

Conditions B (CGA, CTV, CIT, MPA, OTA, RL2, RUG, SAF, VRA): column Agilent Zorbax Eclipse Plus C18 (50 × 2.1 mm, 1.8 µm), gradient elution (A: 5 mM ammonium formate, 0.2% formic acid, pH 3, B: methanol/2-propanol, 9/1, v/v; min-%B-flow) 0–30-0.4, 0.1–40-0.4, 8–60-0.4, 10.5–60-0.4, 11–90-0.4, 12.5–100-0.4, 12.51–100-0.56, 14–100-0.56, 14.5–30-0.56, 29.5–30-0.56, 30–30-0.4, 40 °C, injection volume 20 µL.

### Method validation

Limits of detection (LOD) and quantification (LOQ) were determined following the paired observations approach as described in EUR 28099 EN [46]. For this purpose, 10 matrix blank filters were split into two equal halves. While one-half of each filter was directly subjected to sample preparation and LC–MS/MS analysis, the other half was spiked with a standard mix meeting the expected LODs prior to extraction. Due to the reduced filter size, extraction was performed with 1.5 mL of methanol/2-propanol (9:1, v/v) instead of 3 mL. In addition, 8-point equidistant calibration curves not exceeding tenfold the expected LOD were prepared in duplicate using two moderately loaded matrix blank filter extracts (see Electronic Supplementary Material, Section S-5 for further details). Calculation of LODs and LOQs followed Eqs. 1 and 2, where *s*_y,net_ corresponds to the standard deviation of the net signals of paired observations and *b* to the slope of the calibration curve.

1 $${\text{LOD}}=\text{5.2}\cdot \frac{{\text{s}}_{\text{y,net}}}{\text{b}}$$

2 $${\text{LOQ}}=\text{3.3}\cdot {\text{LOD}}$$

Linearity and working ranges of both LC–MS/MS methods were subsequently derived in triplicate from 10-point equidistant calibration curves in the range of 1 to 30–80 × LOQ in matrix blank extracts. Following linear regression and statistical analysis (Mandel’s fitting test [47], Neumann’s trend analysis [48]), linear working ranges were defined for every analyte (see Electronic Supplementary Material, Section S-6 for further details). For determination of accuracy, two matrix blank filters of comparable moderate loading were split into six parts each, spiked at LOQ level, and subjected to sample preparation (methanol/2-propanol, 9/1, v/v; 0.5 mL) and LC–MS/MS analysis. In accordance to appropriate guidance documents, accuracy encompasses both trueness (how close the result is to the true value) and precision (how consistent the results are). As no certified reference material was available, recovery defined as the proportion of the analyte recovered after sample preparation was used as an estimation of trueness in this context [49]. Intra-day values were obtained on day 1 (*n* = 6), while inter-day values were derived from paired samples on day 1, 3, and 5 (*n* = 3 × 2).

## Results and discussion

Sampling of dust for mycotoxin analysis has mostly been carried out in working environments where dust is an issue by nature, like bakeries, farms, or waste treatment facilities [50, 51]. Instead, investigations of infested construction materials or moldy dwellings are much less prominent [7, 14], probably because occupational safety measures do not apply to renting or households in general. With an ongoing debate whether sampling should focus on settled or airborne dust [52], we focused on airborne dust as we were interested in exposure due to inhalation. Although inhalational exposure may be extrapolated from findings relating to floor dust, an increase in mycotoxins in airborne particulate matter of up to 15-fold has been reported compared to bulk material [53]. While these findings related to storage of cereal grains, a similar effect may be observed comparing total suspended particulate matter to settled dust leading to an underestimation of inhalable mycotoxin concentrations. Additionally from a more technical perspective, the “cleaner” sample matrix of TSP compared to settled dust recovered from household cleaning products is another benefit [54, 55].

**Fig. 1:**
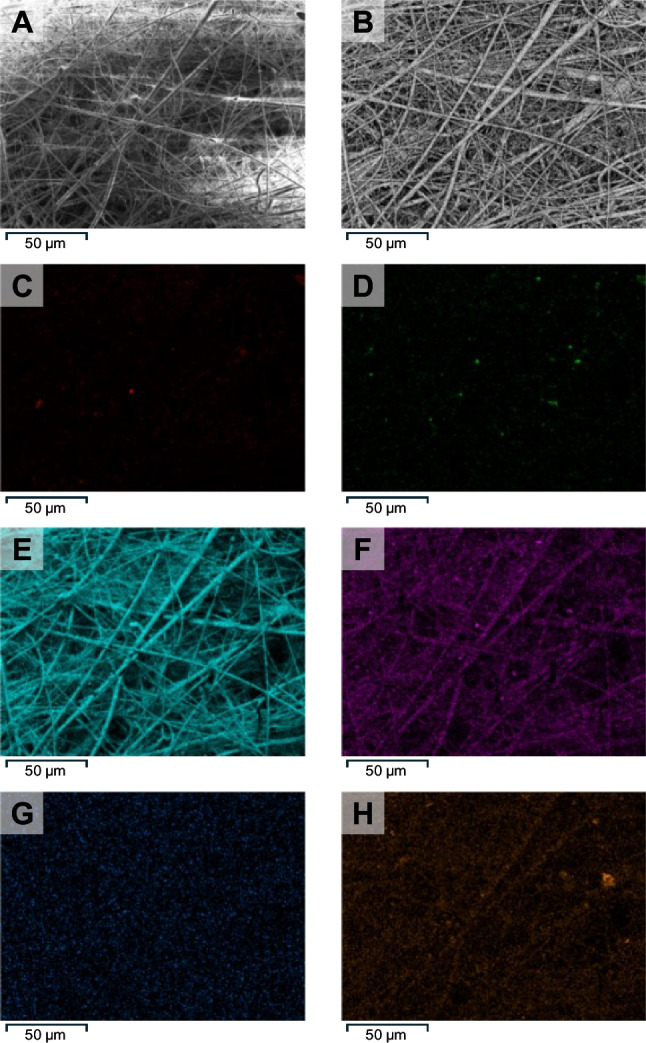
Scanning electron microscopy (SEM) of a densely loaded filter at 500 × magnification. Airborne dust particles are barely visible but can be distinguished as small objects along the glass fibers (**A**). Using electron backscatter diffraction (EBSD), organic matter appears dark and inorganic matter like mineral components or metallic particles appears gray to white (**B**). Energy-dispersive X-ray (EDX) analysis detects organic matter by the presence of carbon (**C**) and nitrogen (**D**). Oxygen (**E**) matches the glass fiber structure as observed by SEM, while sodium (**F**) and chlorine (**G**) are present as part of the glass fibers as well as sweat particles. Aluminum (**H**) was detected within the glass fibers and as aluminum oxide particles

Technical details of how to collect the relevant inhalable fraction of particulate matter have already been elaborated and standardized [56], which is why the main work presented here starts with TSP sample collection. Here, using a uniform set of matrix blanks for method development and validation is an important but not easy to achieve prerequisite. The following outlines the characterization of matrix blank filters used in this study. Out of a total of 34 filters obtained from one sampling area, four filters ranging from low to high loading were analyzed by SEM/EBSD. As shown in Fig. 1A, B, dust particles mainly occurred as a mixture of organic and inorganic matter, where organic matter appears dark and inorganic matter gray to white. Metallic particles were also identified, but they were sparse and notably small. Detailed analysis of each loading’s composition was subsequently achieved by EDX providing distribution maps of each element as shown in Fig. 1C–H. While mappings of oxygen and silicon match with the glass fiber structure of the filters, mappings of carbon and nitrogen showed scattered intense spots indicating organic particles like dandruff, tallow, or hair fragments. Elements like sodium and chlorine typically present in sweat also co-localized nicely and were scattered similar to carbon and nitrogen. Sodium and aluminum were detected in the glass fibers but with less intensity when compared to spots representing dust particles, the latter probably originating from aluminum profiles often used in door sills. As composition of individual loadings was considered comparable based on the observed relative and visual elemental distribution, classification was subsequently carried out with respect to the number of particles deposited (see Electronic Supplementary Material, Table S-7.1 for details on relative elemental composition).

Mapping all 34 filters using reflected-light microscopy from the outer edge to the center in several layers and subsequent superposition provided grayscale images with all particles displayed in sharp focus. Histographic analysis using ImageJ [44] revealed differences mainly in the shade range from 0 to 155 (see Electronic Supplementary Material, Section S-7 for further details). With most of the filter loadings within a range of 1 to 20%, exclusion of the filters below 1% provided a large group of 28 moderately (1.1–21.4%) and 3 heavily (30.3–100%) loaded filters, which were used for method development and validation.

Based on previous reports analyzing airborne particulate matter for mycotoxins like AFB1, STC, OTA, zearalenone, and several macrocyclic trichothecenes [13, 57], chromatographic separation was first attempted in one run. Albeit testing several combinations of different stationary and mobile phases, merging all 29 mycotoxins into one single method with acceptable peak shape and separation failed. The tested chromatographic conditions included fully porous as well as core–shell C18 columns of different lengths, phenyl-hexyl, and pentafluorophenyl (F5) stationary phases. They were combined with organic eluents, such as acetonitrile, methanol, or a methanol/2-propanol mixture (9/1, v/v), as well as aqueous phases that were adjusted to different pH values. Chromatography of tenuazonic acid was especially challenging due to its notable acidity, which required shifting pH to above 9 [58].

To account for possible adverse pH effects on some of the other analytes, chromatographic separation was finally carried out by two separate methods using 5 mM ammonium formate and methanol/2-propanol (9/1, v/v) at either pH 3 on a Zorbax Eclipse Plus C18 column or pH 10 on a Gemini NX-C18 column. In addition, sample extracts and chromatographic eluents were premixed just before chromatography using the autosampler of the LC system and silanized inserts used in the beginning were replaced by in-needle-mixing to reduce consumption of laboratory supplies.

**Fig. 2:**
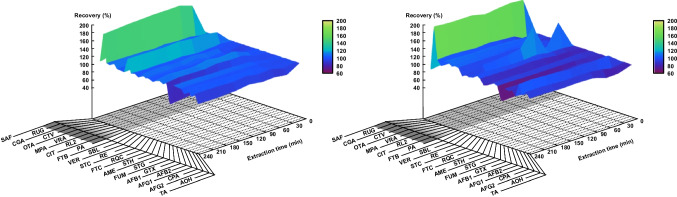
Extraction kinetics using an unexposed (left) and a moderately loaded (right) filter (methanol/2-propanol (9/1, v/v), 20 °C, sonication). Recoveries were calculated based on a standard mixture in extraction solvent (shown at 0 min extraction time). Increased recoveries were observed for RUG (180–204%) and CIT (113–140%), while FUM was the only analyte showing a significant recovery decrease over time down to 60%. Mycotoxins analyzed according to chromatographic conditions B are shaded in gray

After having achieved efficient chromatographic separation, each parameter for mass spectrometric detection was carefully optimized, followed by a thorough evaluation of conditions for mycotoxin extraction from the loaded filters. Methanol/2-propanol (9/1, v/v) was again used to avoid a negative influence on chromatographic separation or peak shapes, and extraction by sonication at ambient temperature was studied for 30, 60, 90, 120, and 240 min, calculating recoveries with respect to a standard mixture in extraction solvent. Using an unexposed filter first, stable recovery rates were observed in the range of 81–126% except for RUG and CIT, showing 178–190% and 131–138%, respectively (Fig. 2, left; see Electronic Supplementary Material, Table S-8.1 for further details). Matrix effects by co-extracted filter components leading to ionization enhancement are considered to cause this behavior. FUM was the only mycotoxin with a decline in recovery over time down to 57% when using an unexposed filter.

Comparable results were obtained when the experiments were repeated using a moderately loaded blank matrix filter (Fig. 2, right; see Electronic Supplementary Material, Table S-8.2 for further details). RUG and CIT again showed high recoveries of 196–204% and 114–122% corroborating previous findings. Interestingly, SBL showed varying recoveries between 103 and 178%, likely resulting from a scattered contamination of the applied blank matrix filter during sampling with spores of *Stachybotrys* spp., which may be present indoors to some extent. As the filter had to be split into parts to six replicates, single spores may have caused higher SBL concentrations in individual fractions. FUM again showed a recovery decrease over time. When extracting a heavily loaded filter for 30 and 60 min, RUG and CIT showed even higher recovery rates of 356–365% and 158–166%, whereas all other mycotoxins remained at 88–124% (see Electronic Supplementary Material, Table S-8.3 for further details). While recoveries for CIT did not change significantly with increased matrix load and are likely caused by the filter material, recoveries for RUG appear to be matrix-dependent and may originate from lipophilic matrix components co-eluting with RUG. No significant variation of SBL recovery was observed, supporting the hypothesis of a scattered contamination as described above. As extended extraction did not provide increased recovery rates but led to a decrease in FUM, an extraction time of 30 min was subsequently applied.

Comparing these findings to literature reports reveals the high variability of the matrix “dust” with slightly to strongly increased signals for selected mycotoxins in TSP in contrast to a matrix-dependent decrease in floor dust [59]. Adequate compensation techniques are thus required for reliable quantification, but lacking isotopologues of the less common mycotoxins required for stable isotope dilution analysis, all quantitative experiments were subsequently carried out by the method of standard addition. Using the autosampler of the LC system for preparation allowed both saving precious reference standards by a factor of up to 80 and minimizing additional error by dilution.

Table 3 summarizes the results from the subsequent validation study. LOD and LOQ determination followed the paired observations approach as described in EUR 28099 EN [46]. Cutting the matrix-loaded filters into halves allowed spiking one-half to the expected LOD while the other half served as a blank of possible native contamination. The observed LOQs in the range of 0.02–34.8 ng/mL (filter extracts) or 3.1–4520 pg/Nm^3^ (sampled volume) fit to reported mycotoxin concentrations in indoor air (Table 1) and are considered suitable for assessment of mold-infested environments. In comparison to literature reports of LC–MS/MS-based multi-mycotoxin detection, the method presented here achieves an LOQ reduction by a factor of 10 to 200 [15, 60]. Intra- and inter-day recoveries determined at LOQ level were in the range of 98 − 129% and 96–129% with corresponding precision values below 20%. These results are very satisfying in respect of ultra-trace analysis, and slightly elevated recoveries (> 100%) are considered acceptable with respect to the intended application in health protection. Linear regression and statistical analysis (*P* = 99%) using Mandel’s fitting test [47] as well as Neumann’s trend analysis [48] applied to the residuals provided linear ranges for every analyte. In addition, the relative standard deviation of the procedure was calculated and required to be no more than 2.5% (see Electronic Supplementary Material, Section S-6 for further details). Working ranges were finally confirmed, allowing for reliable quantification by standard addition of up to 10–20 × LOQ, referring to analyte concentration in the sample.

**Table 3:** Validation data obtained using blank matrix filters (LOD, LOQ determined by paired observations as described in EUR 28099 EN [46]; intra-day and inter-day accuracy and precision were determined at LOQ level, *n* = 6; linear ranges were derived from calibration curves between 1 and 30–80 × LOQ; see Electronic Supplementary Material, Sections S-5 and S-6 for further details)

	LOD (ng/mL)	LOQ (ng/mL)	LOQ^a^ (pg/Nm^3^)	Intra-day		Inter-day		Linear range (ng/mL)		
				Recovery (%)	Precision (%)	Recovery (%)	Precision (%)			
AFB1	0.0181	0.0598	7.77	119	5	123	6	0.0598	–	4.92
AFB2	0.0414	0.137	17.8	120	7	121	7	0.137	–	10.7
AFG1	0.0296	0.0976	12.7	112	5	115	9	0.0976	–	7.38
AFG2	0.0307	0.101	13.2	127	2	118	7	0.101	–	8.20
AME	0.0166	0.0548	7.12	115	6	116	10	0.0548	–	2.46
AOH	0.510	1.68	219	122	9	124	7	1.68	–	52.5
CGA	0.152	0.502	65.1	114	14	123	12	0.502	–	41.5
CTV	0.153	0.504	65.4	101	12	112	14	0.504	–	41.4
CIT	0.0954	0.315	40.9	112	11	123	10	0.315	–	25.7
CPA	0.0106	0.0348	4.53	116	11	106	17	0.0348	–	2.44
FUM	0.310	1.02	133	119	5	123	6	1.02	–	79.6
FTB	1.54	5.08	659	114	10	125	3	5.08	–	395
FTC	0.0737	0.243	31.6	123	10	122	12	0.243	–	18.7
GTX	0.233	0.769	99.8	121	13	118	17	0.769	–	59.8
MPA	0.0116	0.0382	4.97	103	7	114	15	0.0382	–	3.28
OTA	0.0588	0.194	25.2	101	12	112	14	0.194	–	15.6
PA	0.126	0.415	53.9	123	6	121	4	0.415	–	34.3
RQC	0.00919	0.0303	3.94	129	5	129	5	0.0303	–	2.41
RE	0.0393	0.130	16.9	126	9	119	10	0.130	–	10.4
RL2	2.24	7.40	961	114	15	107	15	7.40	–	607
RUG	10.6	34.8	4520	121	5	123	16	34.8	–	2719
STG	0.372	1.23	159	119	11	113	12	1.23	–	95.6
STH	4.87	16.1	2090	122	7	117	8	16.1	–	1260
SAF	2.61	8.63	1120	98	11	96	11	8.63	–	708
SBL	0.231	0.763	99.1	123	5	129	8	0.763	–	59.1
STC	0.00727	0.0240	3.12	115	9	120	3	0.0240	–	1.62
TA	5.59	18.4	2390	109	13	118	18	18.4	–	1440
VRA	0.0651	0.215	27.9	109	5	113	4	0.215	–	18.0
VER	2.69	8.88	1150	124	9	119	10	8.88	–	693

^a^LOQs in pg/Nm^3^ refer to standard sampling conditions of 10-h sampling at a flow rate of 2.3 Nm^3^/h

Representative samples were taken at different working environments involving physical exercise of the staff (Table 4). It must be noted that sampled volumes expressed in relation to normal cubic meters (20 °C, 101.325 kPa) will differ from the actually sampled volumes due to deviations in temperature and air pressure. However, concentrations of airborne mycotoxins should always be reported in relation to normal cubic meters to allow for comparison between different measurements, whereas it is absolutely necessary to use the actually sampled volume for calculation of exposure.

**Table 4:** Mycotoxins detected in indoor air/TSP samples obtained from three mold-infested working environments (see Electronic Supplementary Material, Section S-1 for photos of the sampling sites)

	Sample 1	Sample 2	Sample 3
	Training facility Rain-damaged ceiling	Training aircraft Rain-damaged interior	Restoration site Flood-damaged paneling
Mycotoxins > LOQ^a^ (ng/Nm^3^)	AME: 0.0243 (LOQ: 0.0048)	SBL: 0.192 (LOQ: 0.128)	STC: 0.0505 (LOQ: 0.0054)
	CIT: 0.0555 (LOQ: 0.0274)	SAF: 16.2 (LOQ: 1.5)	GTX: 0.984 (LOQ: 0.172)
	SBL: 0.210 (LOQ: 0.066)		SAF: 11.5 (LOQ: 1.9)
	RUG: 24.5 (LOQ: 3.0)		
Sampling time (h:min)	9:59	3:59	2:55
Sampling flow rate (Nm^3^/h)	3.45	4.46	4.56
Sampled volume (Nm^3^)	34.5	17.9	13.4
Sampled volume (m^3^)	37.1	18.6	13.7
Relative humidity (%)	59.4	58.8	47.9
Temperature (°C)	23.2	18.0	21.4
Atmospheric pressure (hPa)	951	970	988

^a^LOQs adjusted according to sampled volumes expressed in normal cubic meters (Nm^3^)

*AME* alternariol monomethyl ether, *CIT* citrinin, *GTX* gliotoxin, *RUG* rugulosin, *SAF* secalonic acid F, *SBL* stachybotrylactam, *STC* sterigmatocystin

Sample 1 was taken on the upper floor of an abandoned office building used for the training of special forces, whose roof had been damaged allowing rainwater to seep in. In consequence, the suspended ceiling and painted concrete walls were visibly moldy and a musty smell was noticeable throughout the building. To account for the size of the infested area, the sampling flow rate was increased to reach a sampled volume of 34.5 Nm^3^ and subsequent LC–MS/MS analysis revealed a contamination with 24.3 pg/Nm^3^ AME, 55.5 pg/Nm^3^ CIT, 210 pg/Nm^3^ SBL, and 24.5 ng/Nm^3^ RUG, which results in a mycotoxin exposure of 135 pg/h AME, 309 pg/h CIT, 1.17 ng/h SBL, and 137 ng/h RUG considering a respiratory minute volume of 100 L/min during maximum physical exercise [61].

Sample 2 was obtained from an aircraft used for training of mechanics. Not being kept in a hangar and exposed to the elements, rainwater had led to mold behind the interior, which at the time of sampling had already been removed. Although only 4 h of sampling time were available, increasing the sampling flow rate to the maximum of 4.5 Nm^3^/h provided a sampled volume of at least 17.9 Nm^3^. LC–MS/MS analysis revealed 192 pg/Nm^3^ SBL and 16.2 ng/Nm^3^ SAF, which results in a mycotoxin exposure of 333 pg/h SBL and 28.1 ng/h SAF with an estimated respiratory minute volume of 30 L/min during light to moderate mechanic work.

Sample 3 was acquired from a flood-damaged data center undergoing restoration. The water level had remained approximately 40 cm above ground for several days, and especially the lower parts of the wooden paneling were deeply covered with mold, while the flooring had already been removed. Sampling for 3 h at a flow rate of 4.6 Nm^3^/h allowed obtaining a sampled volume of 13.4 Nm^3^. 50.5 pg/Nm^3^ STC, 984 pg/Nm^3^ GTX, and 11.5 ng/Nm^3^ SAF were detected by LC–MS/MS analysis, which results in a mycotoxin exposure of 147 pg/h STC, 2.87 ng/h GTX, and 33.5 ng/h SAF with an estimated respiratory minute volume of 50 L/min for the workers on site.

Since LOQs depend on the sampled air volume, they were individually calculated for each sample based on the actual collected volume in normal cubic meters. The detected mycotoxin levels exceeded the respective LOQs by a factor of 2 to 10. Comparison with previously published mycotoxin levels in airborne dust from water-damaged buildings throughout Europe (Table 1) revealed a good agreement for STC and *Stachybotrys* toxins like SBL [13–15]. In general, our findings were in good agreement with the previously published pattern of toxins prevalent in floor dust, except for our observation that satratoxins and roridins were not detected alongside SBL, which often co-occurs with AME and STG in floor dust samples [55, 62]. However, analyte occurrence varies from region to region and depends heavily on the mold species involved. Furthermore, a direct comparison of airborne to floor dust levels is impossible as TSP samples range in the microgram scale and cannot be accurately weighed.

In summary, these results demonstrate the method’s applicability and sensitivity in quantifying indoor-relevant airborne mycotoxins down to the picogram per normal cubic meter range with LOQs covering concentrations relevant to mold-infested working environments. Although daily inhalation exposure in the lower microgram range may seem low compared to foodborne ingestion, the absence of hepatic first-pass metabolism will contribute to chronic health effects.

## Conclusion

As molds spread by spores, monitoring of airborne spores is common practice as part of mold-infested buildings’ restoration, assessing the type and extent of infestation before and successful removal after sanitation. However, mold becoming visible because of sporulation is just one part of the picture because it does not provide any information about the mold’s production of mycotoxins. These mycotoxins, however, do not just stick to walls but become airborne and may be inhaled by those working or living in the infested building. The method reported here extends the toolbox of indoor mycotoxin analysis by a powerful method for TSP. Covering a total of 29 indoor-related mycotoxins with high sensitivity and sufficient range enables a reliable mycotoxin exposure assessment, which was demonstrated in three representative use cases. As the determined mycotoxin concentrations exceeded the respective LOQs by up to a tenfold, the method may well be suitable for mold-infested buildings with only mild or hidden infestations. In future studies, the reported method will be applied to a sample series from mold-infested buildings to provide further proof-of-applicability and to investigate the often-observed mismatch between observed fungi and detected mycotoxins. Results will be reported in due course.

## Supplementary Information

Below is the link to the electronic supplementary material.

ESM 1 (PDF 1.35 MB)

## Data Availability

The authors confirm that the data supporting the findings of this study are available within the article and its supplementary materials.
