# Supplementary material for: From mold to mycotoxins: an LC–MS/MS method for quantifying airborne mycotoxins in indoor environments
Source: Anal Bioanal Chem. 2025 Jul 23;417(20):4637–48. doi: 10.1007/s00216-025-05980-3 (PMC12325524; doi:10.1007/s00216-025-05980-3)
Supplement: Supplementary file 1 — (PDF 1.35 MB) [file 216_2025_5980_MOESM1_ESM.pdf]

Electronic Supplementary Material

From Mold to Mycotoxins: An LC–MS/MS Method for Quantifying Airborne Mycotoxins in Indoor Environments

Wiebke Derz,[†] Paul W. Elsinghorst[*,†,‡]

[†] Central Institute of the Bundeswehr Medical Service Munich, Ingolstädter Landstrasse 102, 85748 Garching, Germany

[‡] Institute of Nutrition and Food Sciences, University of Bonn, Friedrich-Hirzebruch-Allee 7, 53115 Bonn, Germany

[*] **Corresponding author:**

Paul Elsinghorst, German Federal Ministry of Defence, Stauffenbergstraße 18, 10785 Berlin, Germany. E-Mail: paulelsinghorst@bmvg.bund.de

Table of Contents **page**

**S-1**	Images of sampling sites	S2
**S-2**	Sampling procedure	S5
**S-3**	Autosampler program details applied for pre-mixing and standard addition	S6
**S-4**	Transitions, ionization energies and MS source parameters applied for LC–MS/MS analysis	S7
**S-5**	Calibration curves for LOD/LOQ determination	S9
**S-6**	Calibration curves for determination of linearity and working ranges	S11
**S-7**	Filter characterization	S13
**S-8**	Extraction experiments	S16

S-1 Images of sampling sites

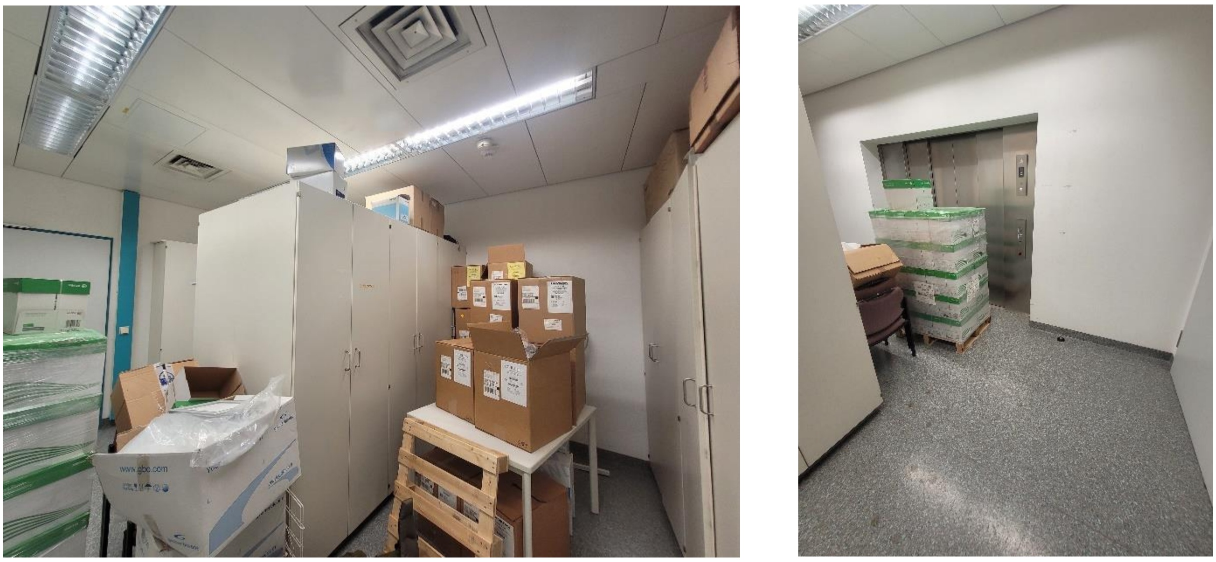

Figure S-1.1 Blank matrix filters were obtained from a dry, visually mold- and odor-free storage room.

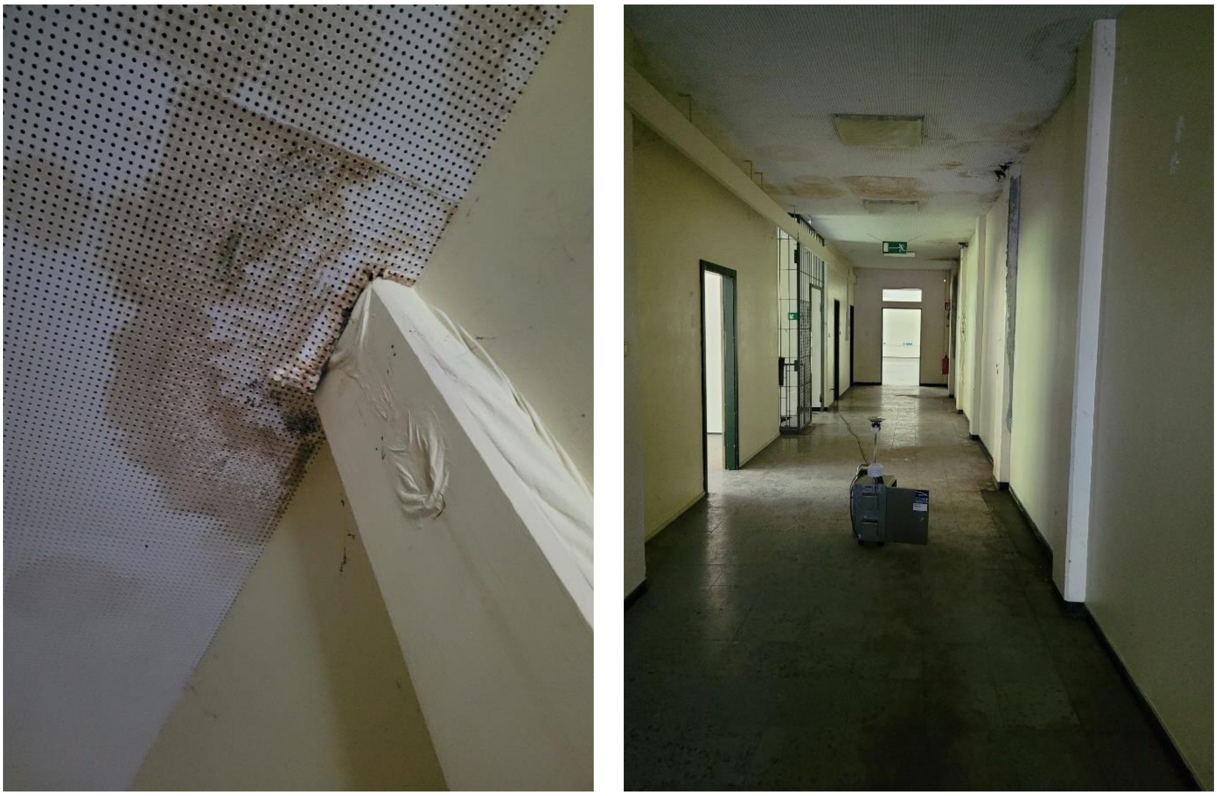

Figure S-1.2 Sample 1 was taken on the upper floor of an abandoned office building used for the training of special forces, whose roof had been damaged allowing rainwater to seep in. In consequence, the suspended ceiling and painted concrete walls were visibly moldy and a musty smell was noticeable throughout the building.

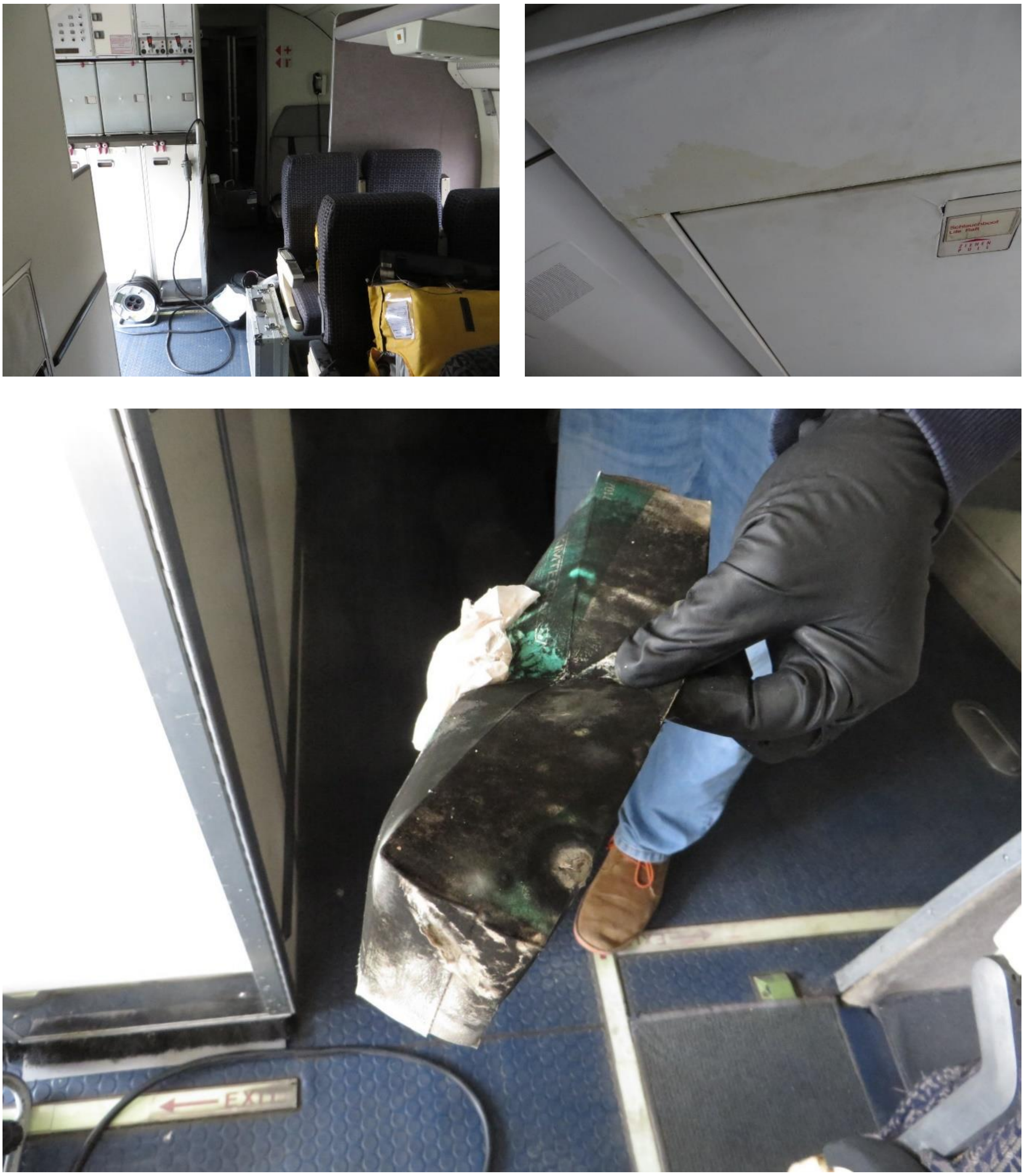

Figure S-1.3 Sample 2 was obtained from an aircraft used for training mechanics. Not being kept in a hangar and exposed to the elements, rainwater had led to mold behind the interior, which at the time of sampling had already been removed except for a tissue box covered in black mold in one of the overhead compartments.

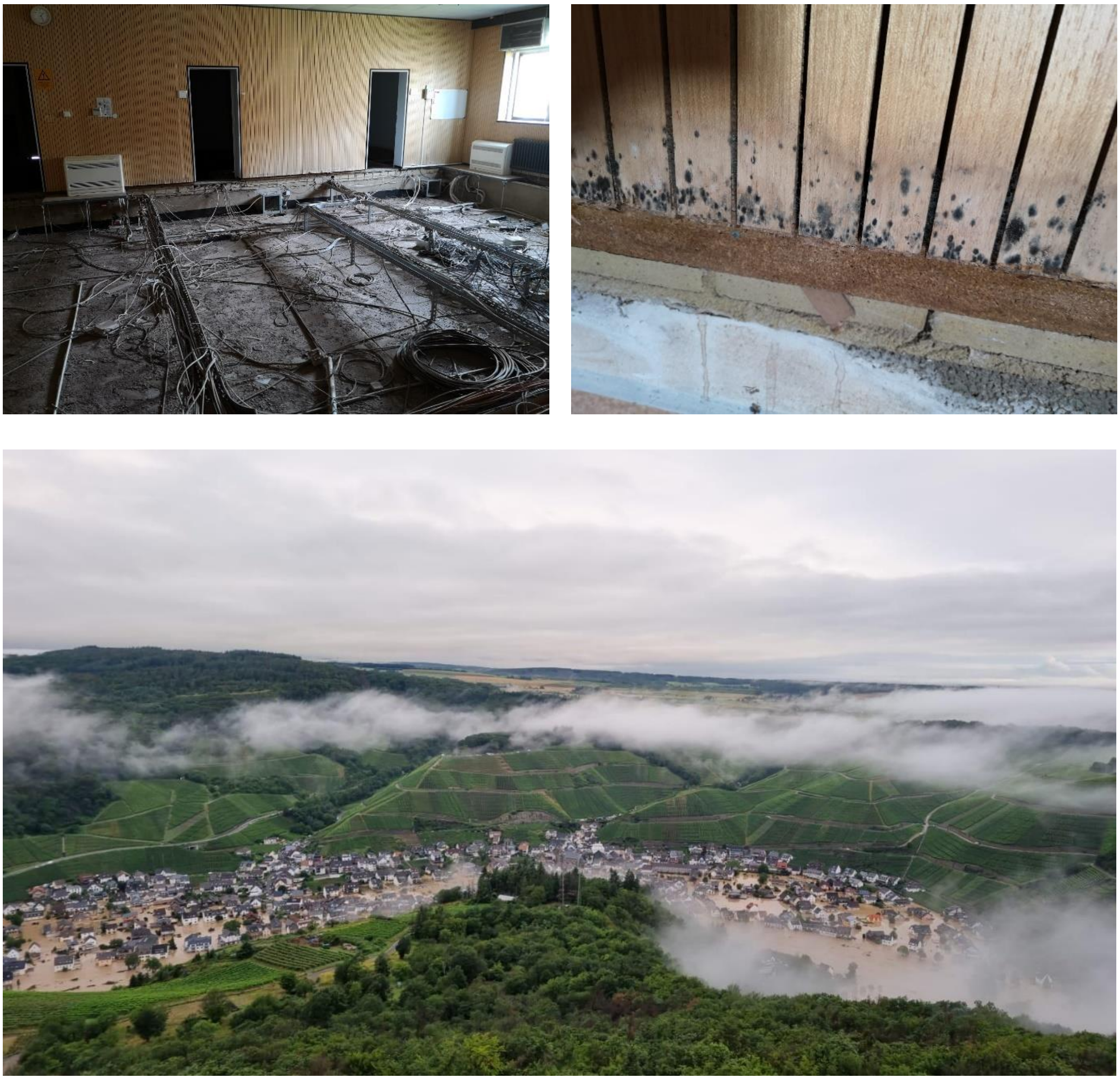

Figure S-1.4 Sample 3 was acquired from a flood-damaged data center undergoing restoration. The water level had remained approximately 40 cm above ground for several days and, especially, the lower parts of the wooden paneling were deeply covered with mold, while the flooring had already been removed.

S-2 Sampling procedure

Air movement was induced by beating out a jacket covering an area of 0.4 m² throughout the sampling site. To ensure systematic coverage, the site was walked across methodically as shown in Figure S-1.1. Every two large steps (approximately 2 meters in total), the jacket was swiftly moved from top to bottom and from the wall toward the center in a single motion. This procedure was repeated along the room's perimeter. In larger rooms, an additional, smaller circular path was followed at a greater distance from the walls. The sampling device (MVS 6.1) was positioned at the center of the room, and air sampling commenced 30 minutes after inducing air movement.

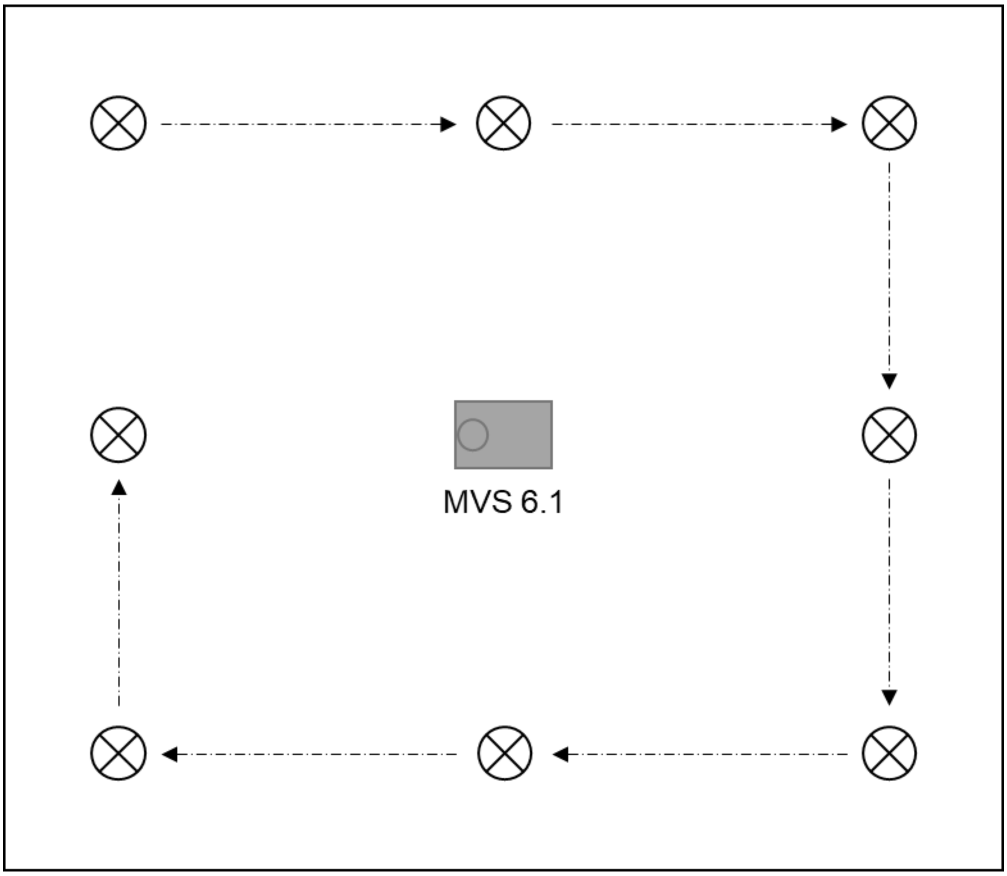

Figure S-2.1 Schematic layout of the sampling site with the air sampler in central position. Markings indicate the positions where indoor air was set in motion prior to sampling.

S-3 Autosampler program details applied for pre-mixing and standard addition

To account for possible adverse pH effects, sample extracts and chromatographic eluents were premixed just before chromatography using the autosampler of the LC system to match chromatographic starting conditions. In brief, calibration standards, eluent (5 mM ammonium formate, pH 3 and pH 10), and vials for washing were prepared and placed in fixed positions of the autosampler. After drawing the required eluent volume (pH 10: 14 µL, pH 3: 24 µL), filter extract (5.4 µL), and calibration standard (0.6 µL, methanol/2-propanol, 9/1, *v*/*v*), in seat mixing was carried out by tenfold repetition (pH 10: 15 µL, pH 3: 10 µL) before injection. To prevent carry-over, needle and sample loop were washed repeatedly using methanol and methanol/water before mixing and injection. The autosampler program was configured as provided in Table S-3.1.

Table S-3.1 Injector program details

Step	Action	Volume (µL)	Draw/Eject Speed (µL/min)
1	Wash needle in flushport for 5 s		
2	Draw & eject methanol	70	1000
3	Draw & eject methanol/water (1/1, *v*/*v*)	70	1000
4	Draw & eject methanol/water (1/1, *v*/*v*)	70	1000
5	Wash needle in flushport for 5 s		
6	Draw eluent A	14 (pH 3)<br>24 (pH 10)	100
7	Draw filter extract	5.4	50
8	Draw calibration standard	0.6	10
9	Wash needle in flush port for 5 s		
10	Mix in seat (10 times)	10 (pH 3)<br>15 (pH 10)	200
11	Inject	20 (pH 3)<br>30 (pH 10)	400

S-4 Transitions, ionization energies and MS source parameters applied for LC–MS/MS analysis

Table S-4.1 Optimized MS source parameters

Parameter	LC conditions A	LC conditions B
Gas temperature (°C)	250	290
Gas flow (L/min)	8	7
Sheath gas temperature (°C)	350	350
Sheath gas flow (L/min)	10	12
Nebulizer pressure (psi)	35	35
Capillary voltage pos/neg (V)	3000 / –3000	3500 / –3500
Nozzle voltage pos/neg (V)	0 / 0	0 / 0

Table S-4.2 MS/MS transitions and optimized parameters for LC conditions A. Precursor ions were $[M+H]^+$ in positive or $[M–H]^–$ in negative ESI mode unless stated otherwise.

Analyte	Retention time (min)	Quantifier<br>Qualifier	CE[a] (V)	Frag[b] (V)	Cell Acc.[c] (V)	ESI mode
Tenuazonic acid	1.8	196.1→139.0<br>196.1→112.0	–20<br>–28	–130	1	neg
Alternariol	2.2	257.1→213.0<br>257.1→147.1	–28<br>–40	–164	1	neg
Cyclopiazonic acid	5.6	337.2→196.1<br>337.2→70.0	24<br>60	116<br>104	1	pos
Aflatoxin G_2	5.9	331.0→189.0<br>331.0→217.0	48<br>40	160	1	pos
Aflatoxin G_1	6.9	329.1→200.0<br>329.1→243.0	48<br>32	146	1	pos
Aflatoxin B_2	7.6	315.0→287.0<br>315.0→259.0	32<br>32	144	1	pos
Aflatoxin B_1	8.4	313.1→285.0<br>313.1→241.0	24<br>44	140	1	pos
Gliotoxin	8.6	327.1→263.2<br>327.1→245.0	8<br>20	78	1	pos
Fumagillin	11.5	459.0→177.0<br>459.0→131.1	16<br>36	110	1	pos
Alternariol mono-methyl ether	12.1	271.1→256.0<br>271.1→227.0	–24<br>–44	–138	1	neg
Satratoxin G	12.3	562.2[d]→249.0<br>545.2→231.0	12<br>20	116	2	pos

Analyte	Retention time (min)	Quantifier Qualifier	CE[a] (V)	Frag[b] (V)	Cell Acc.[c] (V)	ESI mode
Satratoxin H	13.8	529.2→77.1<br>529.2→231.0	128<br>16	136	1	pos
Fumitremorgin C	14.6	380.2→212.2<br>380.2→324.0	40<br>20	130	1	pos
Roquefortin C	16.8	390.2→193.1<br>390.2→322.0	32<br>24	140	1	pos
Sterigmatocystin	17.5	325.0→310.0<br>325.0→281.0	28<br>44	146	1	pos
Roridin E	18.4	532.3[d]→113.0<br>532.3[d]→361.0	28<br>16	116	1	pos
Verruculogen	18.8	512.2→198.2<br>512.2→352.0	24<br>20	108	2	pos
Stachybotrylactam	19.2	386.2→178.1<br>386.2→77.1	40<br>104	196	1	pos
Fumitremorgin B	20.7	480.2→462.2<br>480.2→213.0	12<br>56	108	1	pos
Penitrem A	21.8	634.3→558.0<br>634.3→616.0	24<br>12	164	1	pos

[a] collision energy, [b] fragmentor voltage, [c] cell Accelerator voltage, [d] $[M+NH_4]^+$

Table S-4.3 MS/MS transitions and optimized parameters for LC conditions B. Precursors were $[M+H]^+$ in positive or $[M–H]^–$ in negative ESI mode unless stated otherwise.

Analyte	Retention time (min)	Quantifier Qualifier	CE[a] (V)	Frag[b] (V)	Cell Acc.[c] (V)	ESI mode
Citrinin	4.1	251.0→231.0<br>281.2[e]→249.2	16<br>–20	94<br>–94	1	pos<br>neg
Roridin L2	4.8	531.3→231.0<br>531.3→77.0	24<br>164	106	1	pos
Mycophenolic acid	6.5	321.0→207.0<br>321.0→159.0	24<br>40	76	1	pos
Verrucarin A	7.0	520.2[d]→457.0<br>520.2[d]→231.0	12<br>20	102	1	pos
Ochratoxin A	9.1	404.1→239.0<br>404.1→102.0	28<br>92	106	1	pos
Citreoviridin	9.3	403.0→315.0<br>403.0→297.0	8<br>16	118	1	pos
Chaetoglobosin A	10.4	529.0→130.1<br>529.0→103.1	80<br>128	114	1	pos
Rugulosin	10.6	543.1→273.0<br>541.1→269.0	20<br>–24	128<br>–102	1	pos<br>neg
Secalonic acid F	11.7	639.2→561.2<br>639.2→589.2	28<br>20	144	1	pos

[a] collision energy, [b] fragmentor voltage, [c] cell accelerator voltage [d] $[M+NH_4]^+$, [e] $[M+MeOH–H]^–$

S-5 Calibration curves for LOD/LOQ-determination

Table S-5.1 Concentration ranges and R^2 values of 8-point equidistant calibration curves for determination of LOD/LOQ for LC conditions A.

Analyte	Calibration range (ng/mL)	R^2
Tenuazonic acid	5.39 – 53.88	0.997
Alternariol	0.50 – 5.00	0.998
Cyclopiazonic acid	0.002 – 0.017	0.998
Aflatoxin G_2	0.02 – 0.21	0.996
Aflatoxin G_1	0.03 – 0.31	0.996
Aflatoxin B_2	0.03 – 0.31	0.998
Aflatoxin B_1	0.02 – 0.20	0.995
Gliotoxin	0.18 – 1.78	0.996
Fumagillin	0.36 – 3.64	0.996
Alternariol mono-methyl ether	0.02 – 0.15	0.996
Satratoxin G	0.24 – 2.41	0.998
Satratoxin H	1.84 – 18.39	0.998
Fumitremorgin C	0.05 – 0.46	0.996
Roquefortin C	0.01 – 0.11	0.997
Sterigmatocystin	0.004 – 0.044	0.995
Roridin E	0.01 – 0.12	0.997
Verruculogen	0.53 – 5.28	0.997
Stachybotrylactam	0.09 – 0.92	0.996
Fumitremorgin B	1.73 – 17.29	0.997
Penitrem A	0.13 – 1.25	0.996

Table S-5.2 Concentration ranges and R^2 values of 8-point equidistant calibration curves for determination of LOD/LOQ for LC conditions B.

**Analyte**	**Calibration range (μg/mL)**	R^2
Citrinin	0.07 – 0.65	0.998
Roridin L2	0.46 – 4.58	0.995
Mycophenolic acid	0.004 – 0.038	0.996
Verrucarin A	0.04 – 0.45	0.997
Ochratoxin A	0.03 – 0.32	0.996
Citreoviridin	0.12 – 1.15	0.997
Chaetoglobosin A	0.09 – 0.90	0.996
Rugulosin	6.40 – 64.00	0.997
Secalonic acid F	1.74 – 17.40	0.996

S-6 Calibration curves for determination of linearity and working ranges

Table S-6.1 Linear regression and statistical analysis of 10-point equidistant calibration curves for determination of linearity and working ranges for LC conditions A.

**Analyte**	R^2	**Standard deviation of procedure (%)**	**Mandel's fitting test (critical value, 99% = 12.25)**	**Neumann's trend test for residuals (critical value, 99% = 0.752)**
Tenuazonic acid	0.9996	1.34	0.23	2.73
Alternariol	0.9996	1.47	0.13	2.04
Cyclopiazonic acid	0.9996	1.45	0.44	1.57
Aflatoxin G_2	0.9995	1.48	8.23	1.66
Aflatoxin G_1	0.9996	1.39	3.80	1.21
Aflatoxin B_2	0.9996	1.45	6.83	1.45
Aflatoxin B_1	0.9997	1.18	1.18	1.88
Gliotoxin	0.9996	1.36	4.09	1.71
Fumagillin	0.9996	1.38	0.02	2.57
Alternariol mono-methyl ether	0.9994	1.64	2.02	2.57
Satratoxin G	0.9999	0.55	0.99	2.03
Satratoxin H	0.9998	0.96	6.97	1.40
Fumitremorgin C	0.9998	1.07	1.90	1.43
Roquefortin C	0.9998	0.94	0.68	1.48
Sterigmatocystin	0.9998	1.04	0.06	1.95
Roridin E	0.9996	1.34	0.50	2.12
Verruculogen	0.9998	0.87	0.90	2.35
Stachybotrylactam	0.9996	1.42	0.02	1.64
Fumitremorgin B	0.9999	0.80	0.15	2.37
Penitrem A	0.9996	1.47	0.14	1.74

Table S-6.2 Linear regression and statistical analysis of 10-point equidistant calibration curves for determination of linearity and working ranges for LC conditions B.

**Analyte**	R^2	**Standard deviation of procedure (%)**	**Mandel's fitting test (critical value, 99% = 12.25)**	**Neumann's trend test for residuals (critical value, 99% = 0.752)**
Citrinin	0.9996	1.34	5.49	1.72
Roridin L2	0.9995	1.49	5.11	1.42
Mycophenolic acid	0.9990	2.25	7.35	0.82
Verrucarin A	0.9995	1.58	0.96	2.25
Ochratoxin A	0.9998	0.91	4.68	2.01
Citreoviridin	0.9992	2.01	2.48	1.74
Chaetoglobosin A	0.9997	1.16	1.75	2.22
Rugulosin	0.9995	1.54	0.04	2.90
Secalonic acid F	0.9990	2.33	0.01	1.79

S-7 Filter classification by histographic analysis

Relative elemental compositions of blank matrix filters were calculated based on pixel counts and are provided in Table S-7.1. As expected, oxygen and silicon are the predominant elements because of the glass fiber filter material. Carbon is observed also at higher concentrations showing a slight correlation with visual filter loading, whereas nitrogen is surprisingly below 0.2% for all filters.

Sodium, potassium, magnesium, and calcium as well as aluminum, zinc, sulfur, and iron are also found in low percentage. However, estimation of elemental distribution by EDX is not entirely accurate, especially for elements at low level. As composition of individual loadings was considered comparable based on the observed relative and visual elemental distribution, filters were further classified with respect to the number of particles deposited.

Reflected-light microscopy from the outer edge to the center in several layers and subsequent superposition provided grayscale images of blank matrix filters with all particles displayed in sharp focus (Figure S-7.1). Conversion to grayscale images was subsequently carried out using ImageJ (version 1.54f), which allowed for histographic analysis with inevitable differences in image brightness being equalized by setting the darkest image value to 0 and the brightest to 255. Compared to a blank filter, histographic differences were mainly observed in the shade range from 0 to 155 (Figure S-7.2). Corresponding pixel counts were added up, normalized with respect to the total pixel count and compared to the most densely loaded filter assigned a reference loading of 100%.

Table S-7.1. Loading and elemental composition of different matrix blank filters obtained by scanning electron microscopy (SEM) using electron backscatter diffraction (EBSD), energy-dispersive X-ray analysis (EDX) as well as histographic analysis following reflected-light microscopy.

**Filter**	**1**		**2**		**3**		**4**	
Visual loading	high		medium		medium		low	
Histographic loading	100%		12.1%		10.3%		4.5%	
Magnification	50×	500×	50×	500×	50×	500×	50×	500×
O	42.3	42.7	45.6	40.7	43.0	43.7	43.3	43.8
Si	26.6	28.2	35.6	34.7	32.9	33.1	34.2	34.0
C	14.4	11.4	4.9	5.8	5.1	6.0	4.0	3.6
Na	5.2	5.6	7.1	5.6	6.1	7.3	6.2	6.4
Al	2.9	3.0	< 0.2	3.4	3.4	3.4	3.3	3.4
K	2.6	3.1	3.9	4.1	3.6	3.5	3.7	3.5
Zn	2.2	2.4	< 0.2	2.6	2.6	< 0.2	2.6	2.8
Ca	1.7	2.1	2.3	2.5	2.2	1.9	2.0	2.0
S	1.2	1.0	0.2	0.2	0.3	0.3	< 0.2	< 0.2
Mg	0.3	0.2	0.2	0.2	0.2	0.2	0.2	0.2
F	0.2	0.2	0.3	0.2	0.3	0.2	0.2	0.3
N	0.2	< 0.2	< 0.2	< 0.2	< 0.2	< 0.2	< 0.2	< 0.2

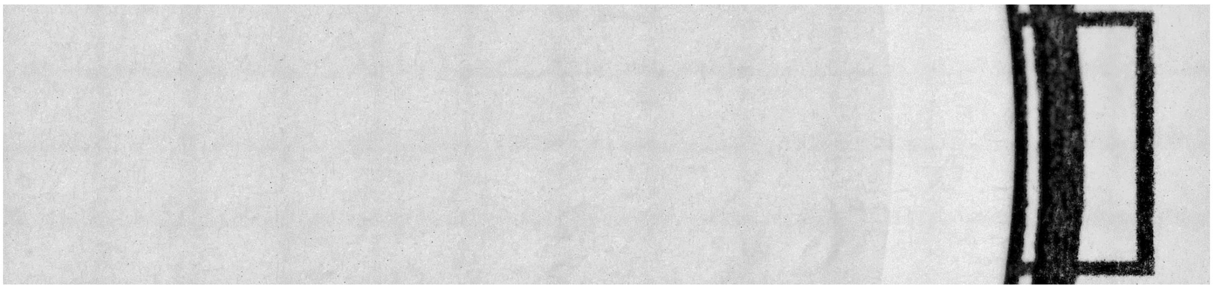

Figure S-7.1 Reflected-light microscopy mapping of an area defined by a black marking on the right (excluded from histographic analysis). The left part of the filter is noticeably more gray because of matrix loading than the almost uncovered white border to the right.

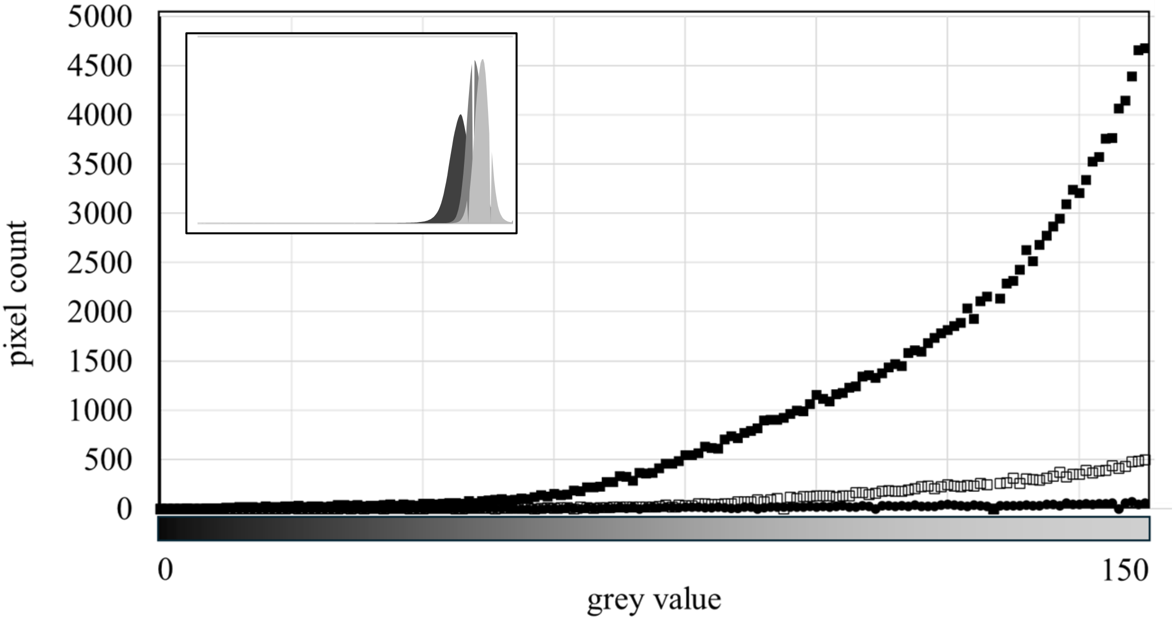

Figure S-7.2 Histographic analysis of filters with high (■), moderate (□) and low (●) loading. Histograms over the entire gray scale range (0 – 255) are shown in the top left-hand corner with the color intensity representing the filter load (■ high, ■ moderate, ■ low).

S-8 Extraction Experiments

Table S-8.1 Recoveries and standard deviations of extraction experiments with an unexposed filter (*n* = 6).

**Analyte**	**30 min**	**60 min**	**90 min**	**120 min**	**240 min**
Tenuazonic acid	81 ± 19	85 ± 21	84 ± 19	88 ± 24	81 ± 20
Alternariol	95 ± 10	104 ± 7	100 ± 11	106 ± 6	103 ± 4
Cyclopiazonic acid	97 ± 4	103 ± 6	109 ± 3	107 ± 4	112 ± 6
Aflatoxin G_2	98 ± 6	101 ± 2	97 ± 4	101 ± 4	96 ± 11
Aflatoxin G_1	94 ± 6	99 ± 3	96 ± 7	97 ± 4	97 ± 2
Aflatoxin B_2	99 ± 5	105 ± 4	105 ± 4	107 ± 7	106 ± 6
Aflatoxin B_1	99 ± 5	105 ± 4	105 ± 7	108 ± 4	107 ± 2
Gliotoxin	96 ± 6	105 ± 6	106 ± 10	108 ± 6	109 ± 7
Fumagillin	88 ± 4	88 ± 5	77 ± 7	74 ± 5	57 ± 24
Alternariol mono-methyl ether	99 ± 7	109 ± 7	107 ± 8	112 ± 6	104 ± 17
Satratoxin G	104 ± 10	114 ± 11	114 ± 13	116 ± 11	110 ± 20
Satratoxin H	105 ± 13	118 ± 15	115 ± 16	119 ± 16	109 ± 27
Fumitremorgin C	103 ± 13	115 ± 15	112 ± 16	115 ± 13	106 ± 24
Roquefortin C	93 ± 4	99 ± 2	99 ± 0	101 ± 3	100 ± 2
Sterigmatocystin	97 ± 4	104 ± 4	103 ± 6	106 ± 2	105 ± 2
Roridin E	83 ± 2	106 ± 4	102 ± 0	110 ± 1	103 ± 2

Analyte	30 min	60 min	90 min	120 min	240 min
Verruculogen	98 ± 13	112 ± 14	111 ± 16	114 ± 14	110 ± 15
Stachybotrylactam	99 ± 4	98 ± 2	98 ± 7	103 ± 2	100 ± 1
Fumitremorgin B	97 ± 18	107 ± 15	105 ± 17	107 ± 13	105 ± 14
Penitrem A	104 ± 1	98 ± 13	100 ± 14	103 ± 12	103 ± 13
Citrinin	131 ± 7	136 ± 3	136 ± 4	138 ± 4	137 ± 3
Roridin L2	97 ± 9	98 ± 5	98 ± 3	102 ± 1	102 ± 4
Mycophenolic acid	106 ± 10	107 ± 8	107 ± 9	113 ± 5	102 ± 12
Verrucarin A	97 ± 9	100 ± 3	100 ± 5	104 ± 5	103 ± 8
Ochratoxin A	111 ± 6	118 ± 3	118 ± 6	121 ± 1	115 ± 5
Citreoviridin	99 ± 5	104 ± 4	104 ± 4	107 ± 3	108 ± 5
Chaetoglobosin A	99 ± 5	105 ± 5	105 ± 6	105 ± 4	109 ± 4
Rugulosin	178 ± 56	188 ± 62	188 ± 46	190 ± 62	182 ± 17
Secalonic acid F	95 ± 27	115 ± 12	115 ± 25	126 ± 22	108 ± 25

Table S-8.2 Recoveries and standard deviations of extraction experiments with a moderately loaded matrix blank filter (n = 6).

**Analyte**	**30 min**	**60 min**	**90 min**	**120 min**	**240 min**
Tenuazonic acid	89 ± 18	88 ± 16	98 ± 19	99 ± 19	100 ± 17
Alternariol	98 ± 21	106 ± 11	111 ± 10	110 ± 9	113 ± 9
Cyclopiazonic acid	125 ± 8	123 ± 7	123 ± 5	123 ± 4	127 ± 8
Aflatoxin G_2	96 ± 3	99 ± 6	93 ± 5	100 ± 8	102 ± 2
Aflatoxin G_1	96 ± 4	98 ± 7	91 ± 4	95 ± 6	93 ± 5
Aflatoxin B_2	103 ± 5	106 ± 4	103 ± 5	107 ± 5	107 ± 4
Aflatoxin B_1	101 ± 3	104 ± 2	101 ± 5	100 ± 6	101 ± 10
Gliotoxin	100 ± 6	103 ± 6	102 ± 7	102 ± 9	104 ± 9
Fumagillin	97 ± 3	91 ± 5	85 ± 6	81 ± 5	68 ± 8
Alternariol mono-methyl ether	103 ± 5	104 ± 4	106 ± 3	108 ± 4	109 ± 5
Satratoxin G	103 ± 8	103 ± 7	103 ± 7	105 ± 9	104 ± 10
Satratoxin H	106 ± 9	105 ± 9	105 ± 9	105 ± 10	104 ± 12
Fumitremorgin C	100 ± 9	100 ± 10	99 ± 9	97 ± 10	98 ± 12
Roquefortin C	90 ± 3	92 ± 2	92 ± 2	92 ± 2	95 ± 1
Sterigmatocystin	102 ± 3	103 ± 4	107 ± 1	109 ± 1	108 ± 5
Roridin E	98 ± 2	109 ± 2	112 ± 2	110 ± 1	115 ± 3
Verruculogen	105 ± 11	105 ± 12	109 ± 9	106 ± 10	105 ± 11
Stachybotrylactam	169 ± 18	107 ± 15	178 ± 26	133 ± 17	103 ± 13

Analyte	30 min	60 min	90 min	120 min	240 min
Fumitremorgin B	109 ± 13	109 ± 10	108 ± 12	105 ± 13	105 ± 14
Penitrem A	101 ± 7	101 ± 5	102 ± 5	100 ± 8	101 ± 7
Citrinin	114 ± 4	122 ± 7	117 ± 4	120 ± 5	119 ± 4
Roridin L2	103 ± 2	118 ± 20	114 ± 13	115 ± 14	119 ± 13
Mycophenolic acid	106 ± 4	110 ± 3	113 ± 5	114 ± 3	116 ± 3
Verrucarin A	104 ± 4	123 ± 24	120 ± 20	123 ± 24	125 ± 22
Ochratoxin A	117 ± 2	116 ± 3	120 ± 4	120 ± 4	122 ± 2
Citreoviridin	105 ± 3	105 ± 9	104 ± 2	109 ± 5	108 ± 5
Chaetoglobosin A	98 ± 4	117 ± 17	118 ± 13	120 ± 16	121 ± 16
Rugulosin	196 ± 14	199 ± 21	197 ± 18	193 ± 16	204 ± 14
Secalonic acid F	99 ± 6	106 ± 8	101 ± 12	94 ± 9	91 ± 8

Table S-8.3 Recoveries and standard deviations of extraction experiments with a heavy loaded matrix blank filter (n = 6).

**Analyte**	**30 min**	**60 min**
Tenuazonic acid	102 ± 5	102 ± 4
Alternariol	88 ± 2	91 ± 1
Cyclopiazonic acid	120 ± 5	123 ± 4
Aflatoxin G_2	104 ± 5	104 ± 4
Aflatoxin G_1	98 ± 4	101 ± 5
Aflatoxin B_2	101 ± 6	100 ± 3
Aflatoxin B_1	99 ± 4	100 ± 4
Gliotoxin	91 ± 5	96 ± 4
Fumagillin	99 ± 3	92 ± 6
Alternariol mono-methyl ether	104 ± 3	106 ± 4
Satratoxin G	100 ± 5	102 ± 6
Satratoxin H	102 ± 7	100 ± 6
Fumitremorgin C	95 ± 7	97 ± 6
Roquefortin C	90 ± 4	92 ± 3
Sterigmatocystin	102 ± 3	105 ± 3
Roridin E	97 ± 4	99 ± 2
Verruculogen	99 ± 9	101 ± 8
Stachybotrylactam	101 ± 5	105 ± 6
Fumitremorgin B	100 ± 11	102 ± 11
Penitrem A	96 ± 7	99 ± 5
Citrinin	158 ± 4	166 ± 7
Roridin L2	99 ± 4	96 ± 3
Mycophenolic acid	115 ± 5	113 ± 3
Verrucarin A	99 ± 3	97 ± 3
Ochratoxin A	122 ± 3	122 ± 3
Citreoviridin	96 ± 3	95 ± 4
Chaetoglobosin A	100 ± 5	100 ± 5
Rugulosin	365 ± 10	356 ± 10
Secalonic acid F	124 ± 6	121 ± 7
